# Efficacy and safety of secukinumab in conjunction with surgery in moderate to severe hidradenitis suppurativa

**DOI:** 10.1016/j.jdin.2026.01.015

**Published:** 2026-02-10

**Authors:** Junyou Zheng, Qingyun Wu, Ke Zhang, Qian Zhang, Qun Lv, Lili Wang, Liming Huang, Yan Wang, Xiangdong Gong, Xiaofang Li, Fang Fang, Wenbo Bu

**Affiliations:** a Hospital for Skin Diseases, Institute of Dermatology, Chinese Academy of Medical Sciences and Peking Union Medical College, Nanjing, Jiangsu, China; b Department of Dermatologic Surgery, Hospital for Skin Diseases, Institute of Dermatology, Chinese Academy of Medical Sciences and Peking Union Medical College, Nanjing, Jiangsu, China; c National Center for STD Control Institute of Dermatology, Chinese Academy of Medical Science, Nanjing, Jiangsu, China; d Department of Medical Mycology, Hospital for Skin Diseases, Institute of Dermatology, Chinese Academy of Medical Sciences and Peking Union Medical College, Nanjing, Jiangsu, China; e Jiangsu Key Laboratory of Molecular Biology for Skin Diseases and STIs, Nanjing, Jiangsu, China

**Keywords:** biologics, combined therapy, hidradenitis suppurativa, secukinumab, surgery

*To the Editor:* Secukinumab, an interleukin-17A inhibitor, improved outcomes in moderate-to-severe hidradenitis suppurativa (HS) in 2 phase 3 trials.^1^ Surgery remains essential for established tunnels, and expert guidance supports integrating medical and surgical approaches in selected patients.^2^ However, real-world evidence on efficacy of secukinumab combined with surgery is limited. We evaluated effectiveness and safety of secukinumab in conjunction with deroofing and/or excision in a retrospective cohort.

Adults (aged ≥ 18 years) with Hurley stage II to III HS treated at our center (2021-2024) were eligible if they received secukinumab plus local/wide deroofing and/or excision and had complete 52-week follow-up. Secukinumab was given as 300 mg weekly for 5 weeks, then every 4 weeks thereafter. Timing varied: 13 patients initiated secukinumab 3 months preoperatively, 8 during the perioperative period, and the remainder postoperatively. The primary endpoint was change in the International Hidradenitis Suppurativa Severity Score System^3^ from baseline to week 52, assessed by anatomical domain (head/face/neck, axillae, groin, and buttocks/anogenital). Secondary end points were Hidradenitis Suppurativa Clinical Response (HiSCR),^4^ site-specific HiSCR (Local HiSCR), and patient-reported outcomes (Visual Analogue Scale pain, Dermatology Life Quality Index, and Work Productivity and Activity Impairment Questionnaire [General Health v1.0]).

Seventy patients were included (Table I). Most participants were men (81.4%) with mean age 28.8 ± 9.8 years, and 74.3% had Hurley stage III disease. The cohort was clinically challenging: most had prior antibiotic exposure and some had inadequate response to adalimumab. At week 52, results showed that mean International Hidradenitis Suppurativa Severity Score System decreased significantly across all anatomical regions (all *P* < .001, Fig 1). Overall, HiSCR was achieved by 65/70 patients (92.9%); rates were similar in single-site and multisite diseases (93.5% vs 91.7%; *P* = .65). Local HiSCR ranged from 85.7% to 87.8% across regions (Supplementary Table I, available via Mendeley at https://data.mendeley.com/datasets/stg3trtwm8/1). Patient-reported outcomes improved from baseline to week 52: Visual Analogue Scale pain 7.2 ± 1.7 to 1.5 ± 1.2, Dermatology Life Quality Index 19.8 ± 5.6 to 6.1 ± 4.6, and Work Productivity and Activity Impairment Questionnaire [General Health v1.0] 10.8 ± 4.2 to 3.5 ± 2.6 (all *P* < .001, Supplementary Table II, available via Mendeley at https://data.mendeley.com/datasets/xm4hk98k2h/1).

**Table I:** Patient’s demographics and clinical features

Parameters	Values
Sex, *n* (%)	
Men	57 (81.4)
Women	13 (18.6)
Age, mean ± SD, y	28.8 ± 9.8
BMI, mean ± SD, kg/m^2^	26.6 ± 4.1
Disease course, mean ± SD, y	5.1 ± 4.4
Smoking habit, *n* (%)	20 (28.6)
Family history, *n* (%)	6 (8.6)
Hurley stage, *n* (%)	
II	18 (25.7)
III	52 (74.3)
Number of affected areas∗, *n* (%)	
1	46 (65.7)
2	19 (27.1)
3	2 (2.9)
4	3 (4.3)
Comorbidities, *n* (%)	
Hyperuricemia	15 (21.4)
Hyperlipidemia and fatty liver	12 (17.1)
Acne	10 (14.3)
Psychiatric disorder‡	4 (5.7)
Diabetes	3 (4.3)
Hypertension	2 (2.9)
Psoriasis	1 (1.4)
Systemic lupus erythematosus	1 (1.4)
Previous treatment, *n* (%)	
Antibiotics†	59 (84.3)
Glucocorticoid	12 (17.1)
Isotretinoin	21 (30.0)
Adalimumab	13 (18.6)
Surgery ± PDT	23 (32.9)

*BMI*, Body mass index; *PDT*, photodynamic therapy; *SD*, standard deviation.

∗ Head, face, neck, axillae, groin, buttock, and anus.

† Includes topical and oral antibiotics, such as doxycycline, clindamycin, rifampicin, and metronidazole.

‡ Anxiety, depression, and obsessive-compulsive disorder (OCD).

**Fig 1:**
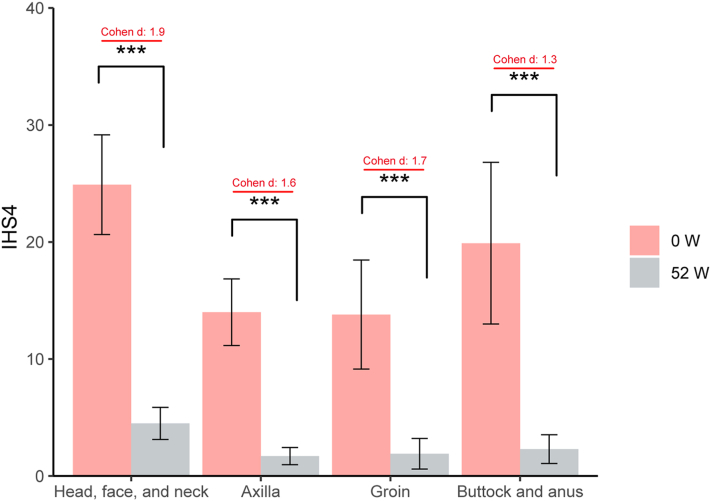
Differences in IHS4 scores of 4 anatomical sites between week 52 and baseline after treatment with secukinumab combined with surgery. ∗∗*P* < .01, ∗∗∗*P* < .001. *IHS4*, International Hidradenitis Suppurativa Severity Score System.

Adverse events occurred in 17/70 patients (24.3%), including 14 considered treatment-related. The most common treatment-related events were upper respiratory tract infection (*n* = 5), HS flares (*n* = 5), and delayed wound healing (*n* = 4). No serious adverse events occurred, and no patient discontinued secukinumab for safety.

Limitations include retrospective design, lack of a control arm, and assessments limited to baseline and week 52. Nonetheless, in a real-world cohort of moderate-to-severe HS undergoing deroofing or excision, secukinumab used in conjunction with surgery was associated with marked clinical improvement and meaningful quality-of-life gains without new safety signals. Perioperative biologic–surgery integration has also been supported in a randomized trial of adalimumab plus surgery.^5^ Prospective controlled studies should define optimal timing around surgery and quantify incremental benefit over either modality alone.

## Conflicts of interest

None disclosed.

## References

[1] Kimball A.B., Jemec G.B.E., Alavi A. (2023). Secukinumab in moderate-to-severe hidradenitis suppurativa (SUNSHINE and SUNRISE): week 16 and week 52 results of two identical, multicentre, randomised, placebo-controlled, double-blind phase 3 trials. Lancet.

[2] Zouboulis C.C., Bechara F.G., Fritz K. (2024). S2k guideline for the treatment of hidradenitis suppurativa/acne inversa-short version. J Dtsch Dermatol Ges.

[3] Zouboulis C.C., Tzellos T., Kyrgidis A., European Hidradenitis Suppurativa Foundation Investigator Group (2017). Development and validation of the International Hidradenitis Suppurativa Severity Score System (IHS4), a novel dynamic scoring system to assess HS severity. Br J Dermatol.

[4] Kimball A.B., Jemec G.B., Yang M. (2014). Assessing the validity, responsiveness and meaningfulness of the Hidradenitis Suppurativa Clinical Response (HiSCR) as the clinical endpoint for hidradenitis suppurativa treatment. Br J Dermatol.

[5] Aarts P., van Huijstee J.C., van der Zee H.H., van Doorn M.B.A., van Straalen K.R., Prens E.P. (2023). Adalimumab in conjunction with surgery compared with adalimumab monotherapy for hidradenitis suppurativa: a randomized controlled trial in a real-world setting. J Am Acad Dermatol.

